# Elderly female CTLs maintain their initial activation and cytotoxicity independent of IL-2

**DOI:** 10.1186/s12979-025-00546-4

**Published:** 2025-11-06

**Authors:** Chantal Hof, Adrian Angenendt, Sandra Janku, Lukas Jarzembowski, Kathleen Seelert, Dorina Zöphel, Markus Hoth, Annette Lis

**Affiliations:** 1 Biophysics, Center for Integrative Physiology and Molecular Medicine, School of Medicine, Saarland University, Homburg, 66421 Germany; 2 Center for Gender-specific Biology and Medicine, Saarland University, Homburg, 66421 Germany; 3 Molecular Biophysics, Center for Integrative Physiology and Molecular Medicine, School of Medicine, Saarland University, Homburg, Germany

**Keywords:** CD8^+^ t cells, Cytotoxic t lymphocytes, Interleukin-2, Cytotoxicity, Ageing

## Abstract

**Background:**

Cytotoxic T lymphocytes (CTLs) are crucial for immune defence, with interleukin-2 (IL-2) playing a central role in their activation, proliferation, and effector functions. However, the impact of IL-2 on CTLs during ageing and across sexes remains poorly understood.

**Results:**

In this study, we investigated the age- and sex-specific influence of IL-2 on CTL activation and cytotoxicity. CTLs from adult mice demonstrated a strong dependence on IL-2 for activation and function, while CTLs from elderly female mice exhibited reduced IL-2 dependence and maintained cytotoxicity under suboptimal IL-2 conditions. This reduction in IL-2 reliance was linked to increased autocrine IL-2 production, enhanced expression of activation markers (CD25, CD69), and effector molecules (perforin, granzyme B). Additionally, elderly female CTLs secreted higher levels of pro-inflammatory cytokines, such as TNF-α and IL-17 A, which may enhance immune responses but also contribute to inflammation if dysregulated. In contrast, CTLs from elderly male mice displayed reduced cytotoxicity and cytokine secretion under low IL-2 conditions.

**Conclusions:**

These findings reveal significant sex-specific adaptations in CTL function during ageing, highlighting that elderly female mice can maintain immune function with less reliance on IL-2. This underscores the need for immune therapies that account for age- and sex-related differences in IL-2 signalling and cytokine regulation.

**Supplementary Information:**

The online version contains supplementary material available at 10.1186/s12979-025-00546-4.

## Background

In recent years, the influence of sex and gender on physiological functions and malfunctions has gained significant attention, particularly during the COVID-19 pandemic. Distinct immunological differences between men and women, along with their tangible clinical consequences, have become evident. Notably, despite comparable or higher infection rates among women [11, 24, 43], men experienced significantly higher rates of hospitalization [9, 51], intensive care admissions [39, 47], and mortality (as reviewed in [43, 53]. These disparities are partly driven by sex-specific differences in immune responses, affecting both innate and adaptive immunity (as reviewed in [13]).

To better understand sex-specific immune differences, animal models are useful and provide critical immunological insights that complement human clinical data. In murine studies, female CD8⁺ T cells have been shown to exhibit increased activation and greater cytokine production, including interferon gamma (IFN-γ) and tumour necrosis factor alpha (TNF-a), compared to males [14, 27]. For example, female mice develop smaller tumours than male [32, 57, 62], a difference attributed in part to enhanced cytotoxic T lymphocytes (CTLs) function. In contrast, male mice exhibit prolonged sickness behaviour following lipopolysaccharide (LPS) exposure [33], while females show delayed recovery following chemotherapy [57]. These observations emphasize that sex-based differences in CTLs responses are biologically meaningful and that murine models can help uncover mechanisms that parallel human immunity, supporting their translational relevance.

CTLs recognize target cells via TCR-MHC-I interactions [64]. Upon activation, CTLs eliminate target cells primarily through granule-mediated cytotoxicity, involving the release of perforin and granzymes to induce target cells death [17, 44]. To terminate immune responses and prevent further activation, CTLs express exhaustion markers, including PD-1, LAG-3 and TIM-3, that modulate their activity [23, 30]. Following infection, a fraction of CTLs persist as memory cells, enhancing the immune response upon re-exposure [21].

Interleukin-2 (IL-2) is essential for T cell regulation [38, 48]. The IL-2 receptor consists of three subunits, namely CD25 (a), CD122 (b), and CD132 (common g chain), which together form the high-affinity IL-2 receptor on CTLs [48, 50, 59]. CD122 and CD132 alone form the intermediate-affinity IL-2/IL-15 receptor [18]. IL-2 signalling activates Janus kinase (Jak) 1 or 3, leading to downstream activation of the signal transducer and activator of transcription 5 (STAT5), phosphoinositide 3-kinases/protein kinase B/mammalian target of rapamycin (PI3K/AkT/mTOR) or mitogen-activated protein kinase/extracellular signal-regulated kinase (MEK/Erk) pathway (as comprehensively reviewed in [61]. Nuclear translocation of various transcription factors induces IL-2-dependent gene expression, regulating components such as CD25 [26] and cytotoxic molecules such as perforin and granzymes [20]. Dysregulated IL-2 signalling has been implicated in autoimmune disorders, including systemic lupus erythematosus, rheumatoid arthritis, and multiple sclerosis (as reviewed in [46]).

While the role of IL-2 on CTL function has been extensively studied, there is limited understanding of how sex influences IL-2 dependent activation, particularly in the context of ageing. Sex- and age-associated immune dysregulation is implicated in conditions such as cancer, chronic infections, and reduced vaccine efficacy, underscoring the importance of examining these factors [15]. In this study, we investigate the impact of sex and age on IL-2 responsiveness and cytotoxic function in murine CTLs. By addressing this critical gap, our findings aim to clarify the interplay between IL-2-signalling and ageing, a key step toward developing potential therapeutic strategies to improve immune function in the elderly.

## Materials and methods

### Mice

Male and female C57BL/6J (wild-type) were purchased from Charles River Laboratories and bred in-house under specific pathogen-free conditions. All animal experiments were approved by local authorities and performed in compliance with the German Animal Protection Law (Tierschutzgesetz, § 11, Abs.1 Nr.1). For the experiments, mice between 14 and 24 weeks (adult) and between 70 and 100 weeks (elderly) were used. Sacrifice was performed by cervical dislocation after CO_2_ intoxication. Mice with splenomegaly or macroscopically visible tumours were excluded. Splenocytes were isolated as previously described [65]. Untouched CD8^+^ T cells were isolated using the Dynabeads™ Untouched™ Mouse CD8 Cells Kit (ThermoFisher).

### Cell culture

CTLs from wildtype mice were cultured in AIM V medium (Thermo Fisher Scientific) containing 10% FCS, 50 µM ß-Mercaptoethanol. Stimulation of wild-type CTLs was achieved by adding Dynabeads Mouse T-Activator CD3/CD28 for T cell Expansion and Activation (Thermo Fischer Scientific) at a 5:4 cell-to-bead ratio. In addition, the specific amount of recombinant human IL-2 (Miltenyi Biotec), 100 ng/mL recombinant human IL-15 (Miltenyi Biotec), and 10 µg/mL Ultra-LEAF™ Purified anti-mouse IL-2 Antibody (Biolegend) was added. Cells were maintained at 37 °C and 5% CO_2_ for three days. P815 cells (ATCC, #TIB-64) were cultured in RPMI-1640 (Thermo Fisher Scientific), supplemented with 10% FCS and 1% penicillin/streptomycin.

### Real-time killing assay

Real-time killing assays were carried out as previously described [31]. Briefly, P815 target cells were loaded with 500 nM calcein-AM in AIM V medium containing 10 mM HEPES for 15 min at room temperature. Cells were washed once and plated at a density of 2.5 × 10^4^ cells/well in black 96-well clear bottom plates (Corning^®^). CTLs from wildtype mice were pulsed with 10 µg/ml anti-CD3ε antibody (Biolegend). Effector cells were gently added onto target cells at a 10:1 effector-to-target ratio. Target cell lysis was measured every 10 min for 4 hours at 37 °C with 5% CO_2_ using a ClarioStar (BMG Labtech) in the bottom reading mode.

### Flow cytometry

All antibodies used for flow cytometry were purchased from BioLegend or Miltenyi Biotec. To determine subtype distribution, stimulated CTLs were stained with PerCP-conjugated anti-CD3, Pacific Blue™-conjugated anti-CD4, FITC-conjugated anti-CD8, PE-conjugated anti-CD44, and APC-conjugated anti-CD62L antibodies. CD122 expression was analysed using FITC-conjugated CD44, Pacific Blue™-conjugated CD62L, and APC-conjugated CD122 antibodies. Samples were acquired on a BD FACSVerse™ flow cytometer (BD Biosciences) and analysed using FlowJo version 10 (FlowJo, LLC).

Activation of CTLs was assessed using Pacific Blue™-conjugated anti-CD8, PE-conjugated anti-CD44, APC-conjugated anti-CD62L, VioBright FITC-conjugated CD25, and PE-Vio 770-conjugated CD69 antibodies. Exhaustion was analysed using APC/Cyanine7-conjugated CD8, PE-conjugated anti-CD44, APC-conjugated anti-CD62L, PerCP-Vio 700-conjugated PD-1, and Brilliant Violet 421™-conjugated CTLA4 antibodies. Samples were acquired on a Miltenyi MACSQuant^®^ Analyzer 16 Flow Cytometer (Miltenyi Biotec) and analysed using FlowJo™ version 10 (FlowJo, LLC).

For combined extracellular and intracellular staining, cells were stained for 20 min with PE-conjugated anti-CD44 and APC-conjugated anti-CD62L antibodies. After washing with PBS/0.5% BSA, cells were fixed in 4% PFA in PBS for 20 min and washed in PBS/0.5% BSA. Permeabilization was performed for 10 min in PBS/0.5% BSA/5% FCS/0.1% saponin/0,007% NaN_3_ and stained with FITC-conjugated Granzyme B and Pacific Blue™-conjugated Perforin antibodies (modified after [28]). Samples were measured on a Miltenyi MACSQuant^®^ Analyzer 16 Flow Cytometer (Miltenyi Biotec) and analysed using FlowJo™ version 10 (FlowJo, LLC).

### Airyscan laser scanning microscopy (LSM)

Intracellular staining for LSM imaging was performed as described before [65]. All the antibodies used were purchased from BioLegend. To identify CTLs and effector molecules, cells were stained with APC-conjugated anti-CD8, Pacific Blue™-conjugated anti-perforin, and FITC-conjugated granzyme B.

High-resolution images of Granzyme B and Perforin in fixed CTLs were taken using an Airyscan-equipped Zeiss LSM880 system. APC- (CD8), FITC- (Granzyme B) and Pacific Blue 410-coupled antibodies (Perforin) were excited using a multi-line argon 488 nm, HeNe 633 nm and diode 405 nm laser lines with 405 and 488/561/633 multi-beamsplitters. Emission light was collected through BP570-620 + LP654 (APC) and BP420-480 + BP 495–550 (FITC, Pacific Blue 410) filters, respectively, and captured using the Airyscan detector. Images were acquired in 16-bit mode with a pixel size of 35–42 nm (at least 1.8x zoom) through a Zeiss Plan-Apochromat 63x/1.4 Oil DIC objective with the pinhole set to at least 1.25 AU (corresponding to the FITC channel). The detector gain was kept between 800 and 900 V and laser power set in such a way that minimal clipping of intensity values within half of the detector range were observed to allow sufficient headroom for image reconstruction (usually < 1% relative power). These settings were set according to the 100U IL-2 condition of female CTLs and kept constant for all following samples. Scans were performed with a pixel dwell time of ~ 1µs and 2x mean averaging. Z-stacks with 0.18 μm intervals throughout the cell were taken for all channels in a sequential manner. Resulting raw Airyscan images from the individual detector elements were aligned, summed and Wiener filtered with the suggested settings in the Zeiss Zen Blue software before being exported. Representative images were imported into Fiji, background-subtracted, reduced in dimensionality by performing a maximum intensity projection and further arranged for displaying purposes using the QuickFigure plug-in.

### CFSE proliferation assay

Proliferation of CTLs from wild-type mice was assessed using CellTrace™ CFSE Cell Proliferation Kit (ThermoFisher) according to the manufacturer’s instructions. CFSE-labelled isolated CTLs were stimulated as described above. Cells were stained with PerCP-conjugated anti-CD8 antibody before acquisition. Cell division of stimulated CTLs was quantified after 48 h on a BD FACSVerse™ flow cytometer (BD Biosciences) and analysed using FlowJo™ version 10 (FlowJo, LLC).

### LEGENDplex™ analysis

Secreted cytokines were analysed using the LEGENDplex™ Th Cytokine Panel (BioLegend). For this purpose, cell culture supernatants of isolated CTLs on day three were prepared according to the manufacturer’s protocol. Briefly, cell culture supernatants were incubated with APC- fluorescent capture beads. Samples were then incubated with biotinylated detection antibodies and stained with PE- coupled streptavidin. Different analytes can be identified due to internal fluorescence and size differences of the capture beads and analyte concentrations can be quantified in relation to the standard curve. Quantification was performed on a BD FACSVerse™ flow cytometer (BD Biosciences) and analysed using the Data Analysis Software Suite for LEGENDplex™ (BioLegend).

### Statistical analysis

Data are presented as mean ± SEM (n = number of experiments). Box-whisker plots display minimum to maximum value (whiskers), 25th to 75th percentiles (box), and median (line). Data were analysed with GraphPad (Prism) software version 9 and 10. For statistical analysis between stimulation conditions, paired ordinary one-way ANOVA was performed in combination with Fisher’s LSD post-hoc test. Significance stars over bar and box-whisker plots indicate significance between sexes as tested by unpaired Student’s t-test. Significance levels were set at *,# *p* < 0.05; **,## *p* < 0.01; ***,### *p* < 0.001 and ****,#### *p* < 0.0001. Asterisks (*) denote differences between conditions, and hash marks (#) between sexes.

## Results

### Diminished cytotoxicity in elderly male mice upon external IL-2 loss reveals contrasting age- and sex-specific responses

**Fig. 1:**
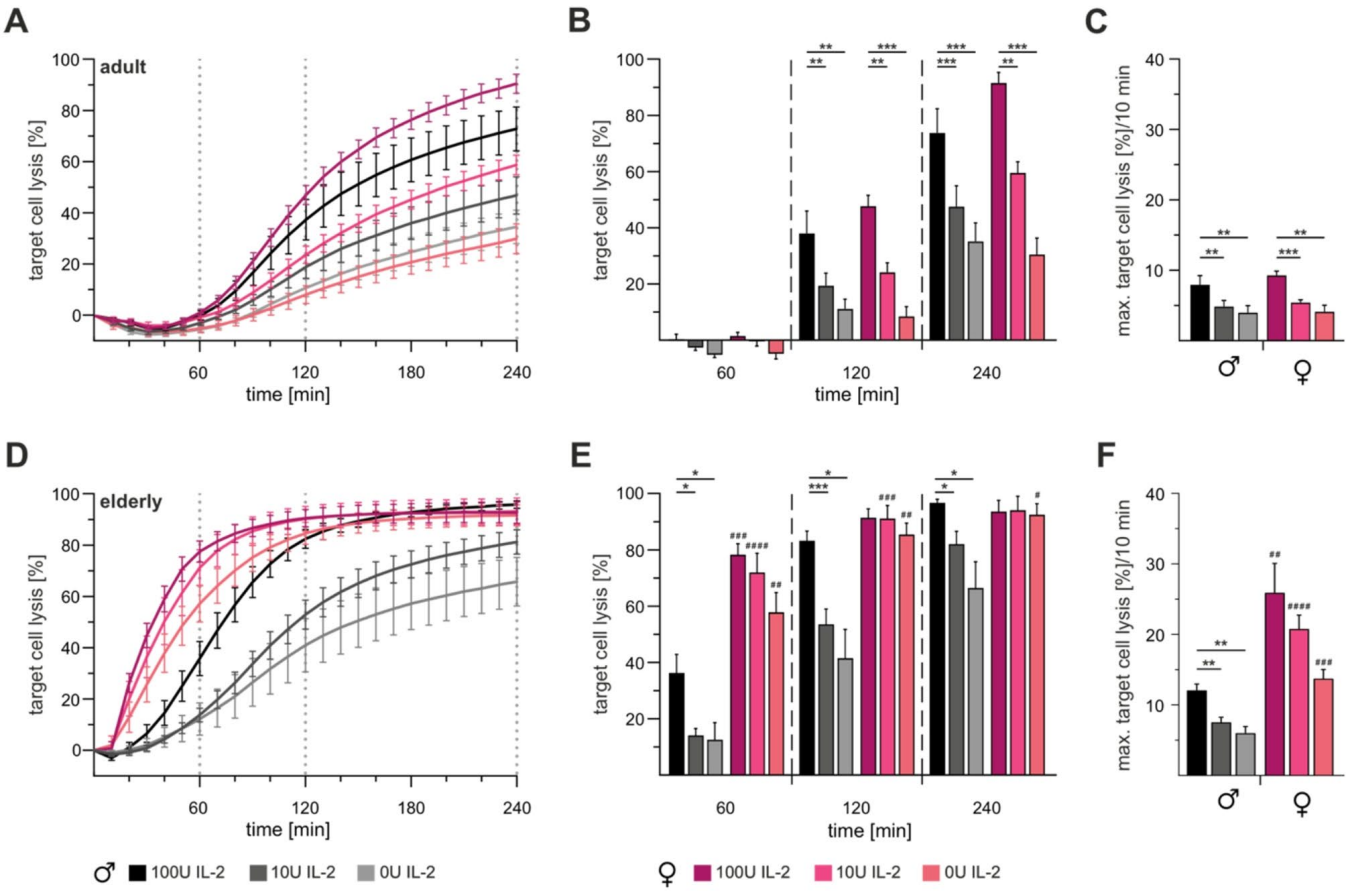
Loss of IL-2 leads to reduced cytotoxicity in adult male and female mice but only in elderly male mice. Real-time cytotoxicity assays were performed on CTLs isolated from adult (**A-C**) and elderly (**D-F**) male (black) and female (berry) mice. CTLs were stimulated under varying IL-2 concentrations (100U, 10U or 0U; shades of black and berry) for three days. The effector-to-target cell ratio was set at 10:1, using P815 as target cells. **B**,** E** Mean percentage of target cell lysis at 60, 120 and 240 min. **C**, **F** Bar plots of maximal target cell lysis per 10 min. Data are presented as mean ± SEM. Significance levels are indicated as *,^#^*p* < 0.05; **,^##^*p* < 0.01; ***,^###^*p* < 0.001 and ****,^####^*p* < 0.0001. Asterisks (*) denote differences between IL-2 concentrations, and hash marks (#) between sexes. Sample sizes were *n* = 5–6 per group

To address a potential role of IL-2 in the context of sex and ageing, we evaluated CTL cytotoxicity from male and female C57BL/6J wildtype mice, comparing adult (Fig. 1A-C) and elderly (Fig. 1D-F) cohorts three days after in vitro stimulation under varying IL-2 conditions (100U, 10U, and 0U). CTLs from both male and female adult mice showed a gradual reduction in cytotoxicity with decreasing IL-2 concentrations (Fig. 1A, B). Target cell lysis over 10 min decreased significantly as IL-2 levels declined, with no discernible sex-specific differences (Fig. 1C).

Consistent with prior observations, CTLs from elderly mice exhibited enhanced cytotoxic kinetics compared to their younger counterparts [65] (Fig. 1A, C). Remarkably, CTLs from elderly female mice maintained consistent cytotoxicity levels throughout the assay, independent of IL-2 concentrations (Fig. 1E). In contrast, CTLs from elderly male mice showed not only reduced kinetics but also diminished endpoint lysis when stimulated with 10U and 0U IL-2 (Fig. 1F). Only with 100U IL-2 exhibited CTLs elderly male mice faster kinetics, which however still remained below those of CTLs from elderly females.

In summary, CTLs from adult female and male mice exhibited comparable reliance on IL-2 for cytotoxicity, independent of sex. However, elderly female CTLs demonstrated a remarkable ability to sustain cytotoxic efficiency under IL-2-depleted conditions, suggesting less dependence on IL-2. By contrast, elderly male CTLs exhibited a pronounced reduction in function under low IL-2 conditions, highlighting a sex-specific disparity in IL-2 dependence with ageing.

### Neutralization of autocrine IL-2 abolishes CTL effector function which is reserved by IL-15 supplementation

IL-2 and IL-15 share two-thirds of their receptor subunits, enabling them to induce overlapping signalling pathways that impact CTL fate. While IL-2 drives effector function, IL-15 supports the survival and proliferation of memory and naïve CD8^+^ T cells [22, 35].

**Fig. 2:**
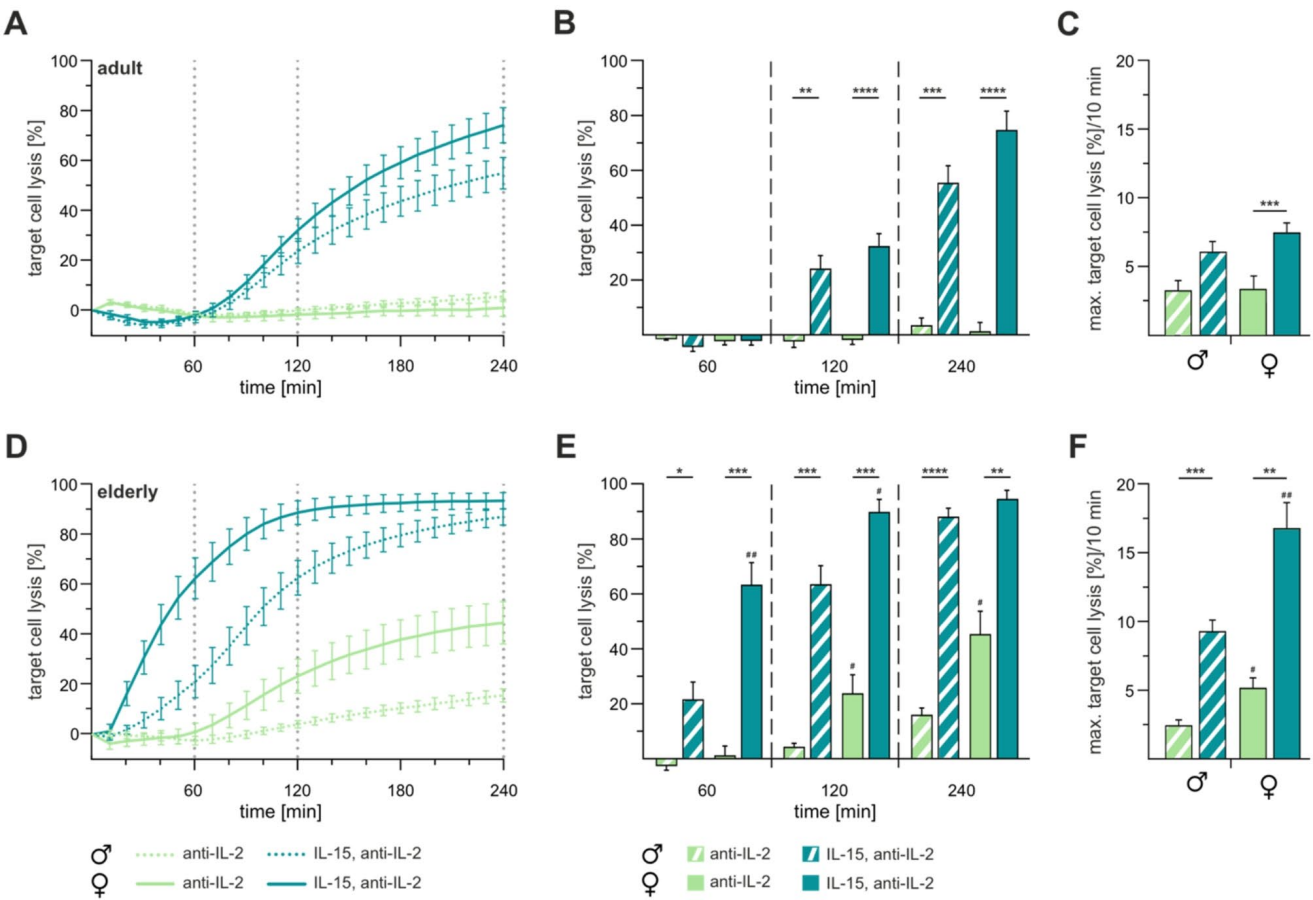
Neutralization of IL-2 abolishes cytotoxicity in CTLs from adult and elderly mice, rescued by IL-15 supplementation. Real-time cytotoxicity assays were conducted on CTLs isolated from adult (**A-C**) and elderly (**D-F**) male (dotted line, patterned column) and female (solid line, filled column) mice. CTLs were stimulated for three days, with anti-IL-2 alone (light green) or with anti-IL-2 + IL-15 (dark green). An effector-to-target cell ratio of 10:1 was utilized, with P815 as target cells. **B**, **E** Mean percentage of target cell lysis after 60, 120 and 240 min. **C**, **F** Bar plots showing maximal target cell lysis per 10 min. Data are presented as mean ± SEM. Significance levels are indicated as *,^#^*p* < 0.05; **,^##^*p* < 0.01; ***,^###^*p* < 0.001 and ****,^####^*p* < 0.0001. Asterisks (*) denote differences between conditions, and hash marks (#) between sexes. Sample sizes were *n* = 5–6 per group

As shown above, CTLs from elderly females retained cytotoxic activity even in the absence of external IL-2 (Fig. 1). To assess autocrine IL-2 production capacity, we employed a neutralizing antibody against IL-2. In adult mice, both male and female CTLs exhibit no cytotoxic activity post-treatment (Fig. 2A, B). Subsequently, IL-15 supplementation was introduced to test whether the intermediate-affinity IL-2/−15 receptor alone could rescue cytotoxicity. The addition of IL-15 significantly enhanced cytotoxicity, restoring activity comparable to that of cells stimulated with 100U IL-2 after 120 min. Although the maximal target cell lysis rate per 10 min was similar between sexes, anti-IL-2 treatment significantly reduced this rate in adult female CTLs compared to their elderly counterparts (Fig. 2C).

In elderly male mice, anti-IL-2 treatment similarly abolished CTL cytotoxicity (Fig. 2D, E). However, CTLs from elderly female mice retained higher effector function, particularly after the 120-minute time point, compared to males (Fig. 2C). Moreover, the maximal target cell lysis rate per 10 min was also less affected by antil-IL-2 in elderly female CTLs compared to male counterparts (Fig. 2F). Notably, IL-15 supplementation restored cytotoxicity in both sexes to levels comparable to those observed in cells stimulated with 100U IL-2 (Fig. 1D, E).

All cytotoxicity assays were conducted using live cell counts to confirm that the observed effects were not due to cell viability (fig. S1). In adult mice, CTL viability decreased significantly with lower IL-2 concentrations and autocrine IL-2 neutralization, from 97% to 85% and 75% in male and female CTLs, respectively (fig. S1A). Supplementation with IL-15 restored viability in both sexes. Similarly, CTLs from elderly male mice showed significant reduction in viability following anti-IL-2 treatment or reduced IL-2 concentrations (fig. S1B). However, addition of IL-15 supplementation significantly increased their viability, even exceeding that observed with 100U IL-2 stimulation. In contrast, CTLs from elderly female mice maintained stable viability across all tested conditions, including anti-IL-2 treatment, further underscoring their reduced dependence on IL-2 during stimulation. Surprisingly, the viability of CTLs stimulated with 100U IL-2 and IL-15/anti-IL-2 in elderly male mice was higher compared to those from elderly female mice, though the differences were not statistically significant (fig S1B).

Together, these finding suggest that elderly CTLs can produce sufficient autocrine IL-2. Moreover, elderly female CTLs appear to possess additional compensatory mechanism that mitigate the effects of external and autocrine IL-2 loss. Finally, IL-15 signalling through the intermediate-affinity IL-2/−15 receptor effectively restores CTL effector function regardless of age or sex.

### Decreasing IL-2 concentrations lead to impaired activation of CTLs

Proper activation is essential for effector functions of CTLs. The activation status of CTLs can be assessed using various markers at distinct activation stages. Initially, CTLs express CD69 on their surface, a marker of early activation. As activation progresses, CD69 expression diminishes while CD25 expression increases [8]. Notably, CD25 expression is directly influenced by IL-2, although TCR signalling alone can also induce its expression [22, 54]. Additionally, fully activated CTLs express high levels of CD122, the β-chain shared by IL-2 and IL-15 receptors [5, 22].

**Fig. 3:**
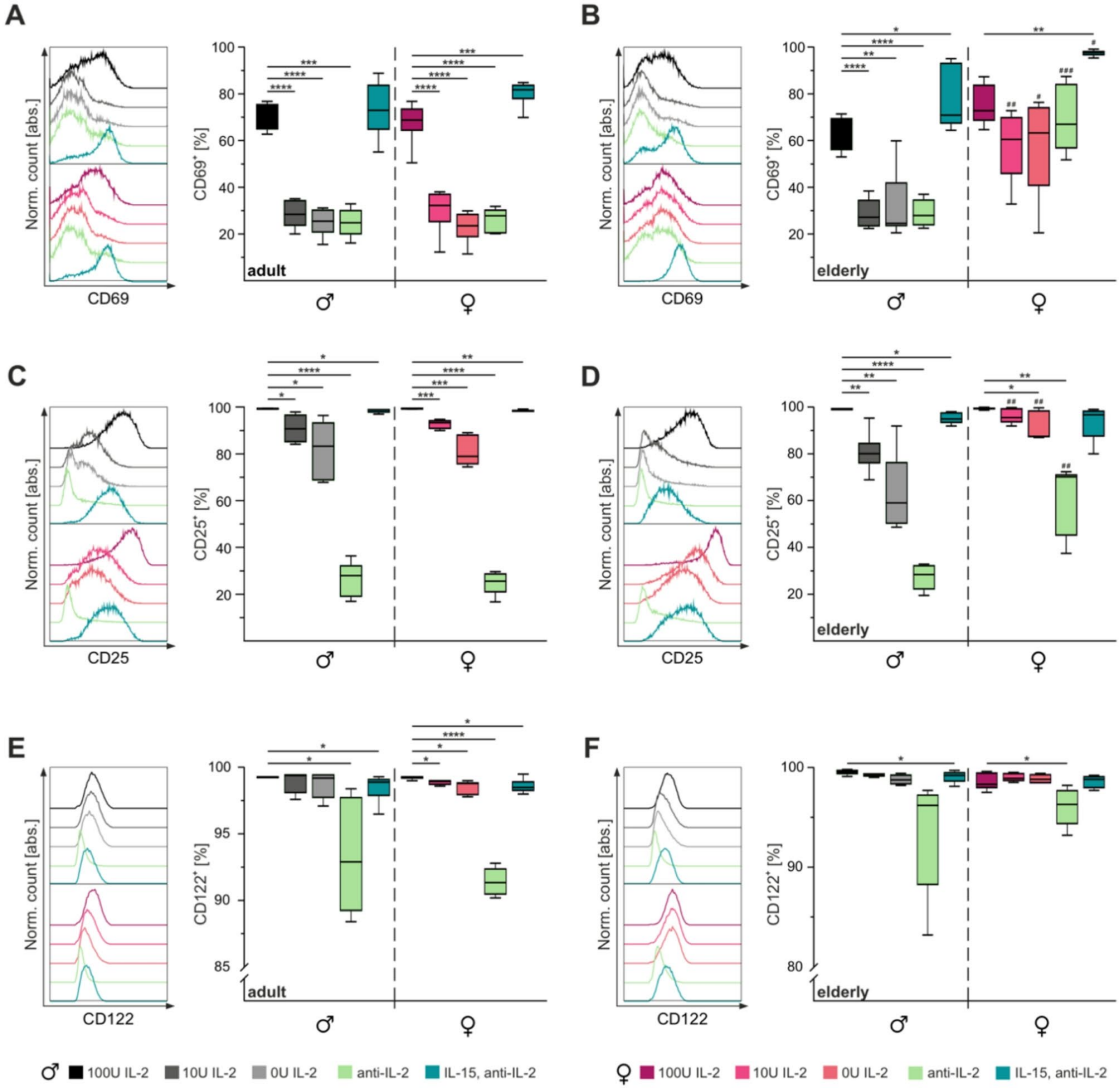
Activation of CTLs from elderly male mice shows stronger IL-2-dependence than those from elderly female mice. Flow cytometry staining of activation markers CD69 (**A**,** B**), CD25 (**C**,** D**), and CD122 (**E**,** F**) on CTLs from adult (**A**,** C**,** E**) and elderly (**B**,** D**,** F**) mice on the third day of stimulation. Single cells were gated for CD3^+^ CTLs. Representative fluorescence profiles (left panel) and corresponding statistical analysis (right panel). Data are presented as median percentage. Significance levels are indicated as *,^#^*p* < 0.05, **,^##^*p* < 0.01, ***,^###^*p* < 0.001 and ****,^####^*p* < 0.0001. Asterisks (*) denote differences between conditions, and hash marks (#) between sexes. Sample sizes were *n* = 5–6 per group

In our experiments, flow cytometry analysis on the third day of stimulation with 100U IL-2 showed that approximately 70% of CTLs were CD69-positive (Fig. 3A, B). Decreasing IL-2 concentrations or neutralization with anti-IL-2 significantly reduced CD69 expression, reaching as low as 25% and 29% in adult and elderly male mice, respectively. Notably, CTLs from elderly female mice retained CD69 expression levels ranging from 58% to 98% across decreasing IL-2 concentrations (Fig. 3B). Treatment with IL-15/anti-IL-2 increased the proportion of CD69-positive CTLs in all groups except adult males.

Unlike CD69, CD25 expression appeared to depend more on the strength of CD3/CD28 stimulation [42]. On day three, at least 93% of CTLs stimulated with 100U IL-2 or IL-15/anti-IL-2 were CD25-positive (Fig. 3C, D). However, CD25 expression decreased significantly with decreasing IL-2 concentrations, particularly in adult mice and elderly male mice, where only ~ 25% of CTLs retained CD25 expression following IL-2 neutralization. Interestingly, elderly female mice exhibited significant reductions in CD25 expression only at 0U IL-2 or after anti-IL-2 treatment (Fig. 3D). Hence, the abundance of CD25-positive CTLs was significantly higher in elderly females than other groups when stimulated with 10U IL-2, 0U IL-2, or anti-IL-2.

Stimulation with 100U IL-2 resulted in > 98% of CTLs being CD122-positive in both adult and elderly mice (Fig. 3E, F). However, the proportion of CD122-positive CTLs decreased significantly in adult females with 10U IL-2 or 0U IL-2 stimulation, and in both sexes following IL-15/anti-IL-2 treatment. Anti-IL-2 treatment caused a significant reduction in CD122-positive CTLS to ~ 91% across all groups, independent of age and sex. Further investigation of CD122 expression using median fluorescence intensity (MFI) revealed similar patterns (fig. S2). CTLs from adult males showed a significant reduction in CD122 MFI under 0U IL-2, anti-IL-2, or IL-15/anti-IL-2 conditions. Similarly, adult female CTLs displayed reduced CD122 MFI at 10U IL-2 (fig. S2A). In elderly mice, male CTLs exhibited reduced CD122 MFI across all conditions, whereas elderly female CTLs displayed stable CD122 MFI except after anti-IL-2 treatment, where CD122 MFI remained significantly higher compared to elderly male CTLs (fig. S2B).

The loss of effector function observed in adult and elderly male CTLs under suboptimal IL-2 stimulation may stem from impaired activation, as indicated by significant reductions in CD69 and CD25 expression, which were exacerbated by anti-IL-2 treatment. In contrast, CTLs from elderly female mice exhibited minimal changes in CD69 and CD25 expression, suggesting a reduced reliance on IL-2 for activation. Interestingly, while CD69 expression persisted in elderly female CTLs treated with anti-IL-2, CD25 and CD122 expression levels decreased similarly to those in male mice. This pattern suggests that elderly female CTLs may remain in an earlier activation phase that is not reached by male counterparts. Despite impaired activation under suboptimal stimulation conditions, minimal changes were observed in CTLs proliferation (fig. S3) or subtype distribution (fig. S4).

### PD-1 expression contrast with CTLA4 expression

As opposed to activation, exhaustion represents a cellular stage characterized by impaired proliferation and effector function, commonly observed during chronic infection and ageing [60]. It can be identified using exhaustion markers such as programmed cell death protein-1 (PD-1) and cytotoxic T-lymphocyte-associated protein-4 (CTLA4). Therefore, we sought to examine the extent to which the decline in cytotoxic activity and the altered sensitivity to IL-2 during ageing are associated with cellular exhaustion.

**Fig. 4:**
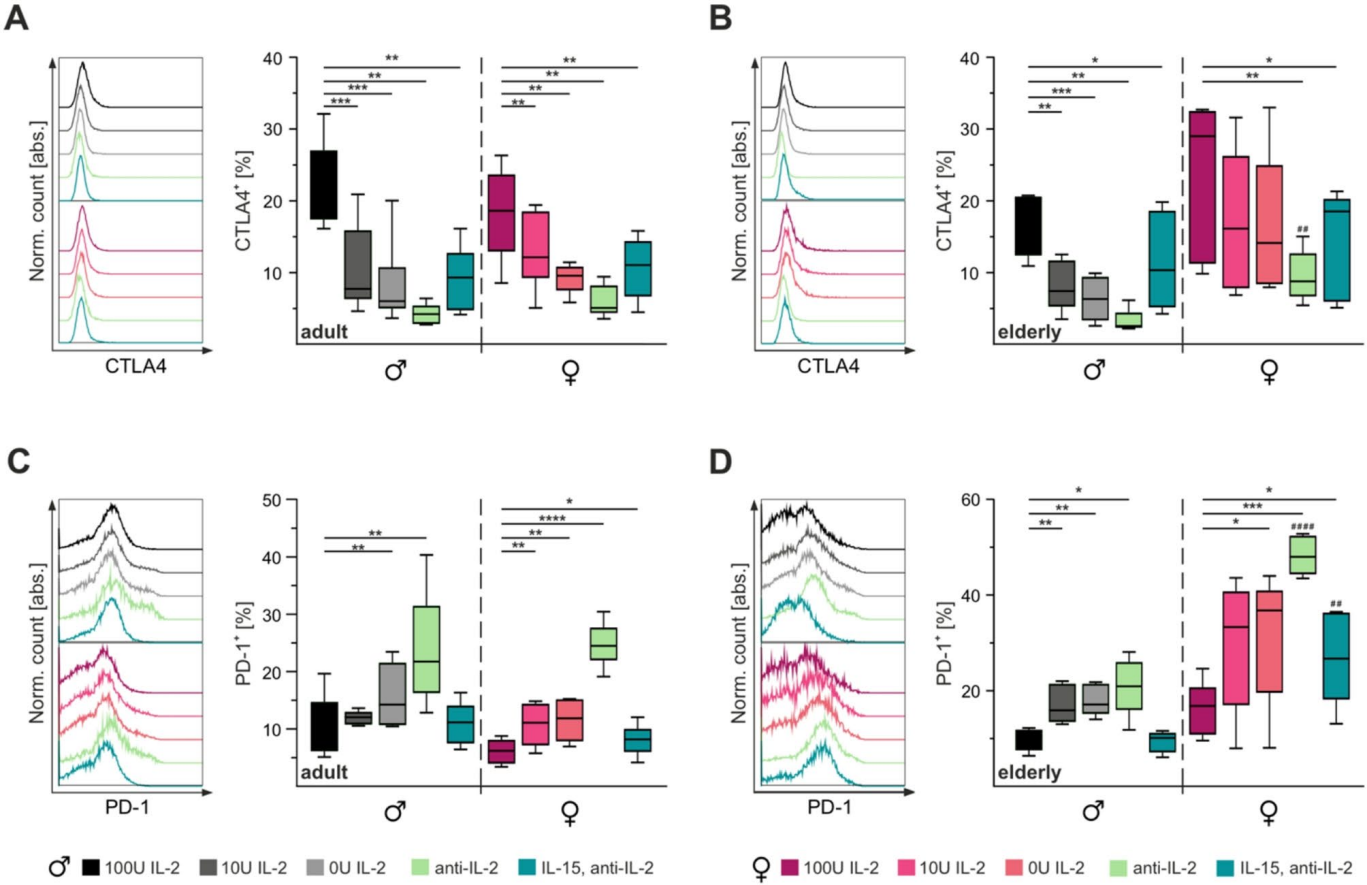
IL-2 dependence of CTLA4 and PD-1 expression. Flow cytometry analysis of exhaustion markers CTLA4 (**A**,** B**) and PD-1 (**C**,** D**) on CTLs from adult (**A**,** C**) and elderly (**B**,** D**) mice on day three of stimulation. Stimulation conditions are represented by different colours. Single cells were gated for CD3^+^ CTLs. Representative fluorescence profiles (left panel) and corresponding statistical analysis (right panel). Data are presented as median percentage. Significance levels are indicated as *,# *p* < 0.05, **,## *p* < 0.01, ***,### *p* < 0.001 and ****,#### *p* < 0.0001. Asterisks (*) denote differences between conditions, and hash marks (#) between sexes. Sample sizes were *n* = 4–6 per group

To investigate this phenomenon, we assessed the expression of PD-1 and CTLA4 on CTLs on day three of stimulation. CTLA4 expression significantly decreased in CTLs from adult mice, regardless of sex (Fig. 4A). Approximately 20% of CTLs stimulated with 100U IL-2 were CTLA4-positive, compared to only ~ 5% in cells treated with anti-IL-2. Similarly, elderly male mice exhibited reduced CTLA4-positive CTLs across all tested conditions (Fig. 4B). In contrast, CTLs from elderly female mice maintained a higher percentage of CTLA4-positive cells, except when stimulated with IL-15/anti-IL-2 or anti-IL-2 alone. When comparing elderly male and female mice, anti-IL-2 treated CTLs from female mice exhibited significantly higher percentage of CTLA4-positive cells than male counterparts.

The percentage of PD-1-positive cells increased as IL-2 concentrations decreased, peaking in cells treated with anti-IL-2, regardless of age and sex (Fig. 4C). In adult male mice, this increase was significant only in CTLs stimulated with 0U IL-2 or anti-IL-2, whereas adult female mice showed a significant increase across all tested conditions. The maximum percentage of PD-1-positive cells in adult mice averaged ~ 25%. CTLs from elderly male mice exhibited significant increases in PD-1-positive cells when stimulated with 10U IL-2, 0U IL-2, or anti-IL-2 (Fig. 4D). In elderly female mice, PD-1 expression significantly increased when stimulated with 0U IL-2, anti-IL-2, or IL-15/anti-IL2, with the latter two conditions resulting in significantly higher PD-1 expression compared to male CTLs. Notably, when stimulated with anti-IL-2, ~ 50% of CTLs from female mice were PD-1-positive compared to only ~ 20% in those from male mice.

In summary, CTLA4 and PD-1 expressions exhibited opposing patterns, with decreasing activation strength leading to reduced CTLA4 expression but increased PD-1 expression. Interestingly, CTLS from elderly female mice displayed either similar or higher expression levels of CTLA4 and PD-1 compared to their male counterparts.

### CTLs from female elderly mice contain significantly more perforin, especially under suboptimal stimulation conditions

Cytotoxicity is directly linked to the expression of effector molecules. In mouse, the most dominant effector molecules are perforin and granzyme B [58]. CTLs from elderly mice have been observed to express higher levels of these molecules compared to their younger counterparts [65]. To investigate whether these differences are sex dependent and are influenced by IL-2 levels, we conducted targeted intracellular staining for perforin and granzyme B.

**Fig. 5:**
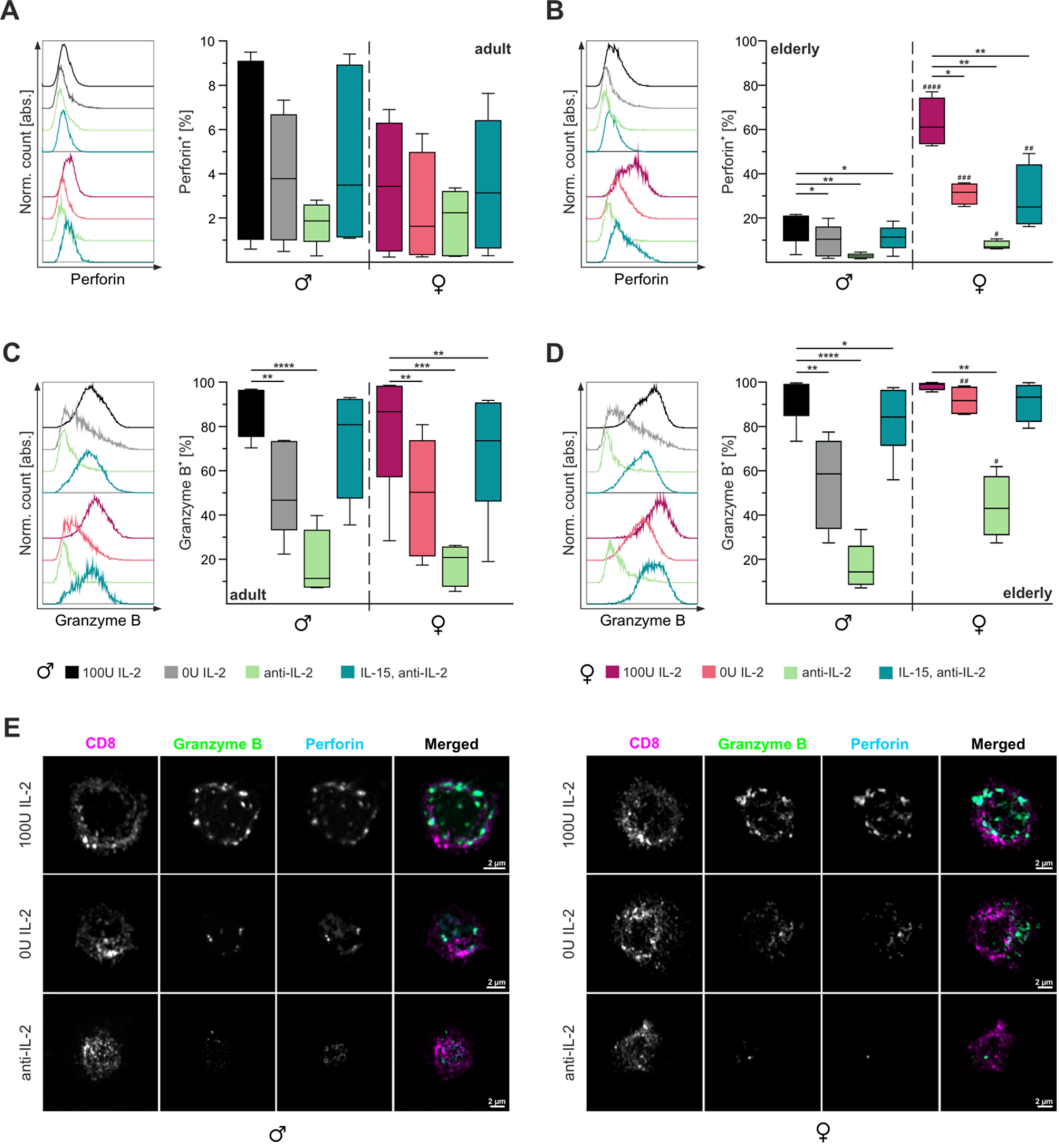
CTLs from elderly female mice show higher abundance of perforin-positive CTLs. Intracellular flow cytometry staining of effector molecules perforin (**A**,** B**) and granzyme B (**C**,** D**) was performed on CTLs from adult (**A**,** C**) and elderly (**B**,** D**) mice on day three of stimulation. Cells were analysed under various conditions, namely 100U IL-2, 0U IL-2, IL-15/anti-IL-2 and anti-IL-2 represented by different colours. Single cells were gated based on CD44/CD62L expression. Representative fluorescence profiles (left panel) and statistical analysis (right panel). **E** Representative Airyscan images of immunofluorescence staining for CD8, granzyme B and perforin in elderly male (left) and female (right) mice. Data are presented as median percentage. Significance levels are indicated as *,# *p* < 0.05, **,## *p* < 0.01, ***,### *p* < 0.001 and ****,#### *p* < 0.0001. Asterisks (*) denote differences between conditions, and hash marks (#) between sexes. Sample sizes were *n* = 5–6 per group

Our analysis showed that in adult mice, the proportion of perforin-positive CTLs was consistently maintained across sexes at different IL-2 concentrations, with no significant reduction observed under anti-IL-2 treatment (Fig. 5A). However, in the elderly mice, we observed a marked decline in the percentage of perforin-positive cells correlating with decreasing IL-2 levels (Fig. 5B). This trend persisted even with IL-15 supplementation (Fig. 5B). Interestingly, a pronounced sex-specific disparity emerged in elderly mice (Fig. 5B, E). Elderly female mice still had ~ 60% perforin-positive CTLs, a rate significantly higher than the ~ 18% observed in their male counterparts, consistent across all experimental conditions.

Granzyme B expression showed a distinct pattern. In adult mice, granzyme B-positive CTLs decreased with diminishing IL-2 concentrations, most notably in females stimulated with IL-15/anti-IL-2 (Fig. 5C). However, this pattern did not display clear sex-specific differences. In contrast, elderly mice showed a significant reduction in granzyme B-positive CTLs, predominantly in males (Fig. 5D). In females, granzyme B levels remained relatively stable, except under anti-IL-2 treatment (Fig. 5D). Notably, CTLs from elderly female mice stimulated with 0U IL-2 or anti-IL-2 exhibited significantly higher percentages of granzyme B-positive CTLs compared to male counterparts (Fig. 5E).

Taken together, these findings suggest that the cytotoxic mechanism in elderly female CTLs remains robust even under low IL-2 conditions, primarily through rapid perforin-induced necrosis. Interestingly, under anti-IL-2 treatment, there appears to be a functional shift toward granzyme B-mediated apoptosis, indicating a flexible cytotoxic response under suboptimal conditions.

### CTLs from elderly female mice secrete higher amounts of TNF-α and IL-2

The cytokines tumour necrosis factor-alpha (TNF-α) and interferon-gamma (IFN-γ) serve as pivotal markers of CTL cytotoxicity. These molecules are critical mediators produced and secreted by CTLs following antigen encounter [58]. To analyse cytokine secretion, we employed LegendPlex™ analysis to quantify different cytokines in parallel in the supernatants of stimulated CTLs.

**Fig. 6:**
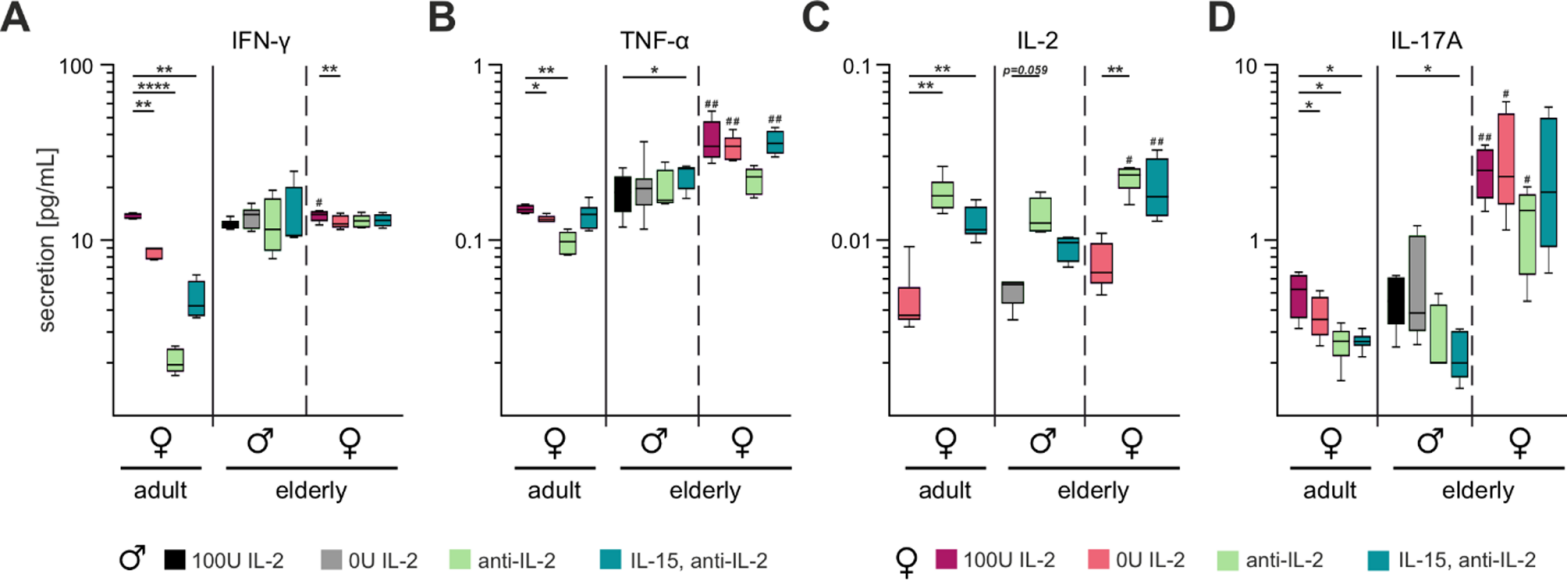
CTLs from elderly female mice secrete higher amounts of TNF-α, IL-2, and IL-17 **A**. LegendPlex analysis of cytokine secretion in cell culture supernatant on day three of stimulation. The figure illustrated the secretion of IFN-γ (**A**), TNF-α (**B**), IL-2 (**C**), and IL17A (**D**) by CTLs. For secretion analysis, supernatants from CTLs of adult female mice (left panel) and elderly mice of both sexes (right panel) were exclusively examined. Data are presented as median concentration. Significance levels are indicated as *,# *p* < 0.05, **,## *p* < 0.01, ***,### *p* < 0.001 and ****,#### *p* < 0.0001. Asterisks (*) denote differences between conditions, and hash marks (#) between sexes. Sample sizes were *n* = 4–6 per group

In adult female mice, IFN-γ secretion by CTLs was highly susceptible to changes in IL-2 concentration, declining significantly from 13.75 pg/mL to 2 pg/mL as IL-2 levels decreased (Fig. 6A). In contrast, CTLs from elderly mice maintained a relatively consistent INF-γ secretion rate, ranging from 12.2 pg/mL to 14.4 pg/mL, regardless of the stimulation conditions. Similarly, TNF-α secretion by adult CTLs was IL-2-dependent, decreasing from 0.14 pg/mL to 0.09 pg/mL under reduced IL-2 levels (Fig. 6B). Notably, IL-15 supplementation restored TNF-α secretion in these cells. Elderly female mice CTLs demonstrated significantly higher TNF-α secretion (~ 0.35 pg/mL) compared to elderly males (~ 0.2 pg/mL). However, under IL-2 neutralization, TNF-α secretion in elderly male and female CTLs was comparable (Fig. 6B).

Activated CTLs can secrete IL-2, although their capacity for secretion is much lower than that of CD4^+^ T cells [52]. In adult female mice, CTLs secreted ~ 0.004 pg/mL IL-2 in the absence of stimulation (Fig. 6C). This secretion increased to ~ 0.018 pg/mL and ~ 0.012 pg/mL under anti-IL-2 and IL-15/anti-IL-2 stimulation, respectively. Elderly male CTLs exhibited a similar IL-2 secretion pattern. However, elderly female CTLs secreted significantly higher IL-2 (~ 0.02 pg/mL) when stimulated with anti-IL-2 or IL-15/anti-IL-2.

IL-17 A, another pro-inflammatory cytokine produced by CTLs, has been implicated in autoimmune diseases such as multiple sclerosis [56], rheumatoid arthritis [40], and psoriasis [63]. IL-17 A secretion by CTLs from elderly female mice remained elevated (1.3 pg/mL and 2 pg/mL) across various stimulation conditions, as shown in Fig. 6D. This secretion profile differed markedly from the reduced one in both, adult females and elderly males. Notably, the lowest IL-17 A secretion was observed in CTLs stimulated with IL-15/anti-IL-2.

In summary, CTLs from elderly female mice exhibit a distinct cytokine secretion profile, characterized by elevated levels of pro-inflammatory cytokines TNF-α, IL-2 and IL-17 A compared to their adult and male counterparts. In the case of IFN-γ, CTLs from elderly males and females displayed comparable secretion levels.

## Discussion

In this study, we observed significant age- and sex-specific differences in murine CTL responses to varying IL-2 concentrations. While adult CTLs from both sexes showed strong IL-2 dependence, elderly male CTLs demonstrated reduced IL-2 dependence, and elderly female CTLs exhibited near-complete IL-2 independence. This distinction is critical, as IL-2 plays a pivotal role in T cell activation, proliferation, and effector function, particularly in immune therapies aimed at enhancing cytotoxicity [45]. Our findings shed light on the unique adaptations of CTLs from elderly female mice, including altered activation dynamics, cytokine secretion profiles, and effector molecule expression, which together maintain robust cytotoxicity even under suboptimal IL-2 conditions.

The IL-2 independence of elderly female CTLs likely arises from their ability to compensate for reduced availability of exogenous IL-2 through autocrine IL-2 production and enhanced reliance on intermediate-affinity IL-2/IL-15 receptors [6]. Increased expression of activation markers (CD25, CD69) and effector molecules (perforin, granzyme B) further supports their higher cytotoxic potential under low IL-2 conditions. Our findings are consistent with prior reports of elevated perforin and granzyme B expression in elderly CTLs [65, 66], as well as higher TNF-α secretion in female CTLs [3]. The pro-inflammatory cytokine TNF-α may serve as a compensatory mechanism for reduced IL-2, as TNF signalling via TNFR2 lowers the TCR signalling threshold and promotes cytotoxic molecule production [4, 25, 34].

Additionally, IL-17 A secretion was significantly elevated in elderly female CTLs, consistent with observations in Tc17 cells. This subset, characterized by IL-17 A, IL-2, TNF-α, and IFN-γ secretion, has been implicated in inflammatory diseases such as Crohn’s disease and inflammatory bowel disease [16, 19]. IL-17 A enhances cytotoxicity by promoting perforin and granzyme B expression, as seen in West Nile virus infection models [1]. Together, these factors contribute to the distinct IL-2-independent functionality of elderly female CTLs.

The ability of elderly female CTLs to maintain cytotoxicity under low IL-2 conditions offers several potential benefits. Robust effector function ensures effective immune defence against infections and tumours, even in ageing organisms where IL-2 production is often impaired [41]. Elevated perforin-mediated necrosis and granzyme B-mediated apoptosis provide flexibility in target cell elimination, while polyfunctional cytokine secretion (TNF-α, IFN-γ, IL-17 A) creates a pro-inflammatory environment that recruits innate immune cells, amplifying immune responses [49].

The sex-specific differences observed in CTL activation and cytokine responses may, in part, be influenced by sex hormones, which are known to modulate immune cell function. Oestrogen and progesterone have been shown to enhance CTLs proliferation and cytokine secretion, while androgens generally exert immunosuppressive effects [27, 55]. Oestrogen signalling can increase IFN-γ and TNF-α production in T cells and promote memory T cell formation, which may contribute to the increased cytotoxicity and cytokine production observed in elderly female CTLs in our study. Although hormone levels decline with age, sex hormone receptor expression persists, and prior hormonal imprinting may shape long-term immune programming. Future studies incorporating hormonal manipulation or receptor profiling may help clarify this link.

However, this enhanced cytotoxicity, and pro-inflammatory state may also predispose elderly females to chronic inflammation and autoimmune diseases. The elevated secretion of IL-17 A and TNF-α aligns with the increased prevalence of autoimmune disorders such as rheumatoid arthritis, lupus erythematosus, and multiple sclerosis in women [27]. Chronic inflammation driven by unregulated CTL activity can contribute to systemic inflammatory conditions like metabolic syndrome or cardiovascular disease [12]. Our findings underscore the delicate balance between protective immunity and immune-mediated pathology in elderly female CTLs.

Interestingly, elderly female CTLs expressed higher levels of exhaustion markers PD-1 and CTLA-4 compared to males, suggesting potential mechanisms to regulate excessive immune responses. While exhaustion is typically associated with functional impairment, such as reduced proliferation and cytokine production [60], our data indicate that elderly female CTLs retain functionality despite PD-1 and CTLA-4 expression. This supports the hypothesis that transient expression of exhaustion markers may correlate with activation strength, acting as a feedback mechanism to prevent immune overactivation [2]. The reduced abundance of PD-L1-expressing antigen-presenting cells in ageing [29, 37] may further limit the functional consequences of exhaustion marker expression in elderly female CTLs.

Our findings highlight the importance of sex- and age-specific considerations in immune therapies targeting IL-2 signalling. The (almost) IL-2–independent functionality of elderly female CTLs suggests that therapies modulating IL-15 or TNF-α pathways may be particularly effective in enhancing cytotoxicity in this population [61]. However, given the already elevated cytotoxic potential and pro-inflammatory profile in elderly females [10], systemic IL-2 or IL-15 administration could carry risks, such as immune overstimulation or increased susceptibility to autoimmunity [7, 36]. This raises important translational considerations: while IL-2– and IL-15–based immunotherapies are feasible in principle, their application in aged individuals — particularly females — may require careful dose titration, personalized cytokine delivery strategies (e.g., tumour-targeted IL-15 prodrugs; [7], and potentially, the co-administration of immune checkpoint inhibitors (e.g., PD-1) to prevent immune-mediated pathology [61]. These findings support a more tailored approach to immunotherapy in the elderly, guided by functional immune profiling rather than age alone.

## Conclusions

In summary, CTLs from elderly female mice demonstrate a unique ability to maintain activation and cytotoxicity under reduced IL-2 conditions, driven by enhanced cytokine secretion and effector molecule expression. While this phenotype provides advantages in immune defence, it also carries potential risks of chronic inflammation and autoimmunity. These findings underscore the need for tailored therapeutic strategies that account for sex- and age-specific differences in IL-2 dependence and immune regulation.

## Supplementary Information

Supplementary Material 1.

## Data Availability

The data supporting the findings of this study are available from the corresponding authors upon written reasonable request.

## Abbreviations

CTL: Cytotoxic T lymphocytes
IL-2: Interleukin-2
PD1: Programmed cell death protein-1
CTLA4: Cytotoxic T-lymphocyte-associated protein-4
TNF-α: Tumour necrosis factor-alpha
IFN-γ: Interferon-gamma
