# Supplementary material for: Elderly female CTLs maintain their initial activation and cytotoxicity independent of IL-2
Source: Immun Ageing. 2025 Nov 6;22:52. doi: 10.1186/s12979-025-00546-4 (PMC12590591; doi:10.1186/s12979-025-00546-4)
Supplement: Supplementary file 1 — Supplementary Material 1. [file 12979_2025_546_MOESM1_ESM.docx]

**Supplemental figures**

**
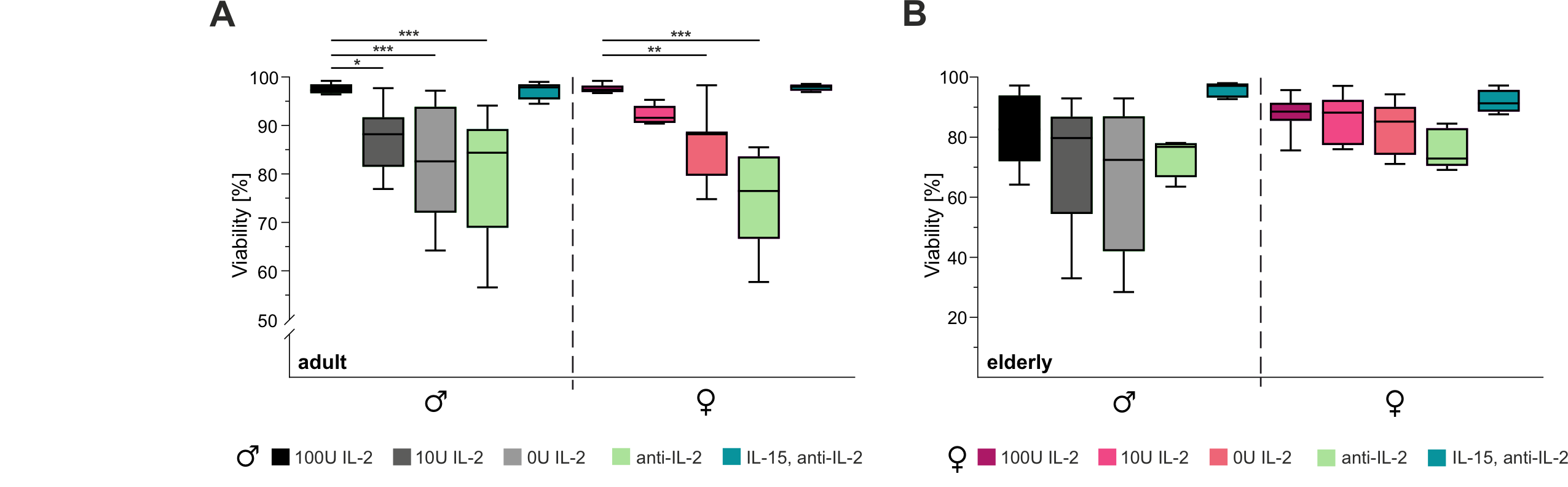
**

**Figure S1:** **Viability of CTLs under IL-2 influence.**  Viability of CTLs from **(A)** adult and **(B)** elderly mice was assessed under varying IL-2 conditions (100U, 10U, 0U) and after treatment with anti-IL-2 antibodies, with or without IL-15 supplementation. Data are presented as median percentage. Significance levels are indicated as * p < 0.05, ** p < 0.01, *** p < 0.001 and **** p < 0.0001. Sample sizes were n = 4-6 per group.


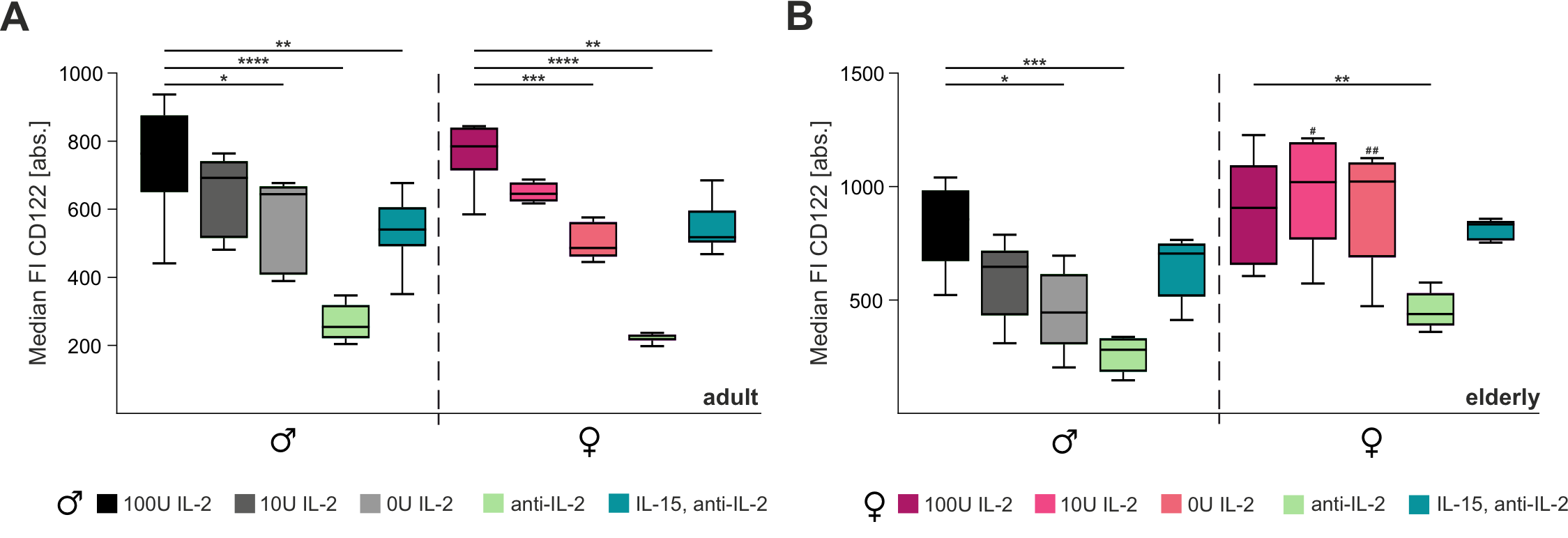


**Figure S2:** **CD122 expression on CTLs under IL-2 influence.** The figure depicts median fluorescence intensities (MFI) of CD122 expression on CTLs from **(A)** adult and **(B)** elderly male and female mice, assessed by flow cytometry following stimulation with varying IL-2 concentrations (100U IL-2, 10U IL-2, 0U IL-2) or treatments such as anti-IL-2 and IL-15/anti-IL-2. Data are presented as median fluorescence intensity (MFI). Significance levels are indicated as *,# p < 0.05; **,## p < 0.01; ***,### p < 0.001 and ****,#### p < 0.0001. Asterisks (*) denote differences between conditions, and hash marks (#) between sexes. Sample sizes were n = 4-6 per group.


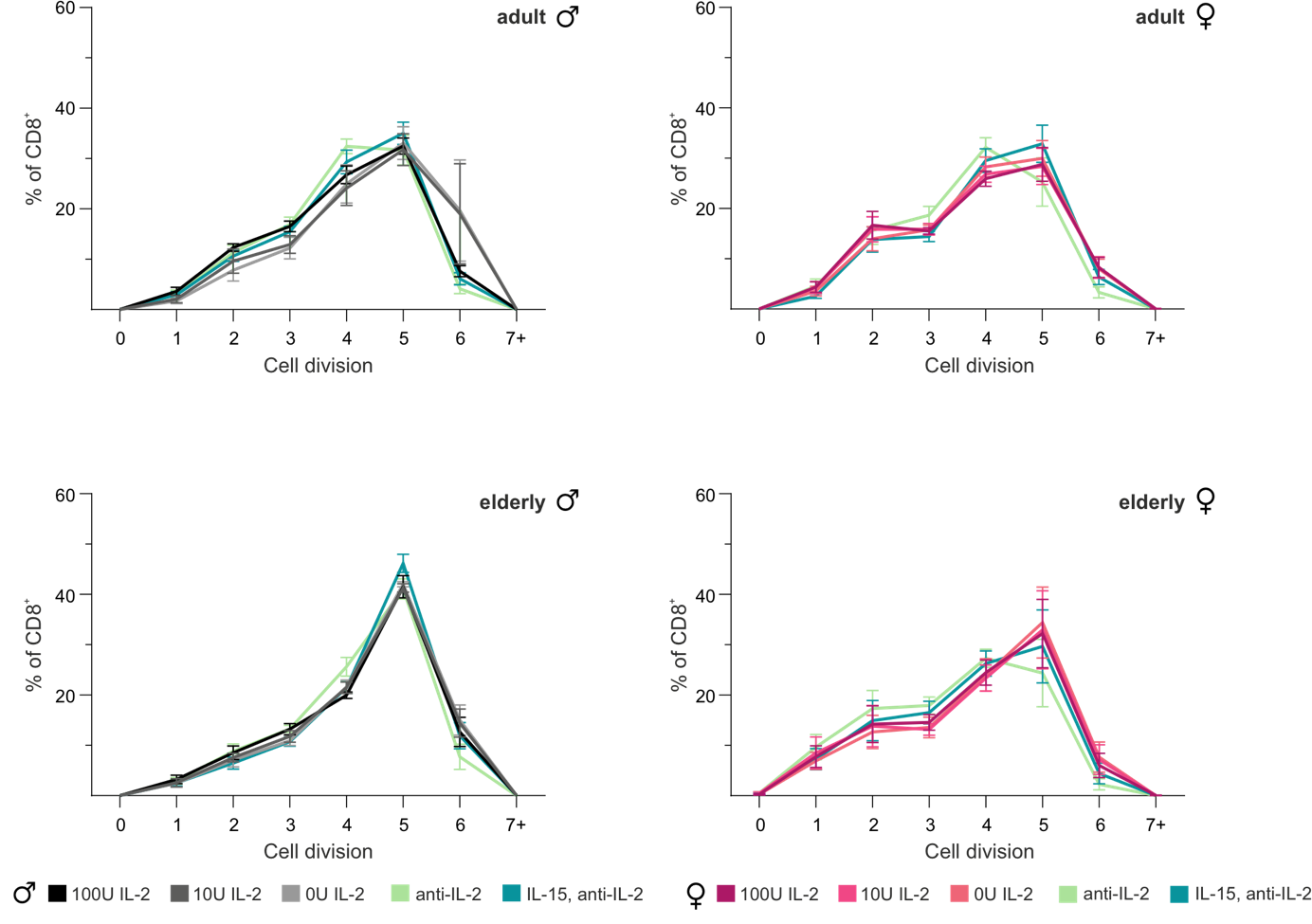


**Figure S3:** **Proliferation of CTLs under IL-2 influence.** The figure shows the proliferation of CTLs across various IL-2 stimulation conditions. Proliferation was assessed using flow cytometry-based analysis of CFSE dilution, which quantifies the quantity of cell division. Panels represent data from adult and elderly mice, respectively. Proliferation rates are depicted for different IL-2 concentrations (e.g., 100U IL-2, 10U IL-2, 0U IL-2) and following stimulation with IL-15/anti-IL-2 or anti-IL-2. Data are presented as mean percentage ± SEM. Significance levels are indicated as * p < 0.05, ** p < 0.01, *** p < 0.001 and **** p < 0.0001. Sample sizes were n = 4-6 per group.


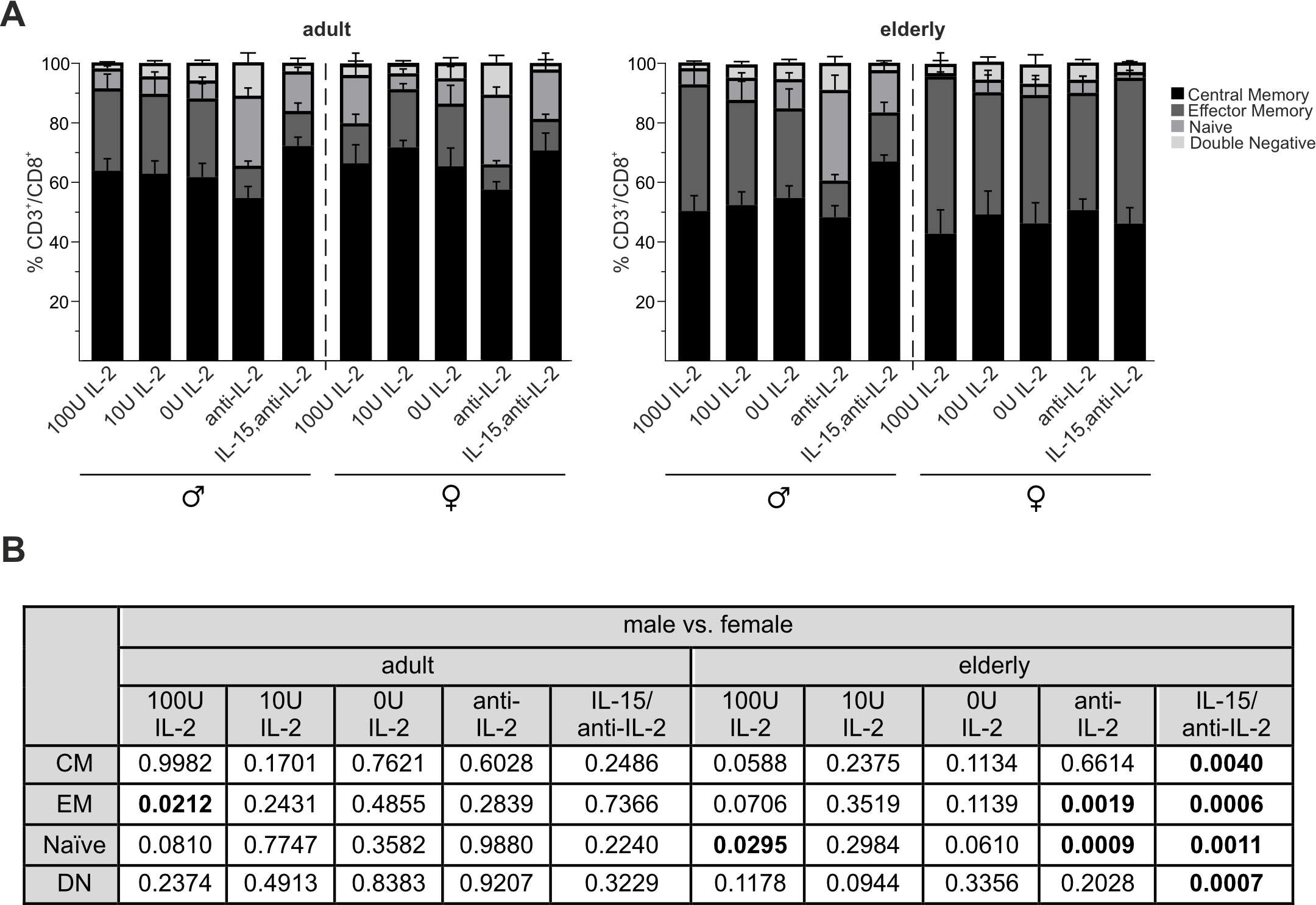


**Figure S4:** **Subtypes distribution of CTLs under IL-2 influence.** The figure depicts the distribution of CTL subpopulations across different IL-2 stimulation conditions. **(A)** Subpopulations are represented by columns, categorized based on CD44 and CD62L expression levels, identifying distinct subsets such as naïve (CD44⁻CD62L⁺), central memory (CD44⁺CD62L⁺), effector memory (CD44⁺CD62L⁻), effector (CD44⁺CD62L⁻), and double negative (CD44⁻CD62L⁻) T cells. Data are presented as mean percentage ± SEM. **(B)** Significance (p-values) between central memory (CM), effector memory (EM), naïve, and double negative (DN) was tested in male against female mice. Significant values (p<0.05) are highlighted. No significant differences were found compared to 100U IL-2. Sample sizes were n = 4-6 per group.
